# Development and Characterization of a Ten-Plex Assay to Measure *Klebsiella pneumoniae* Antigen-Specific IgG in Human Sera

**DOI:** 10.3390/mps8030052

**Published:** 2025-05-19

**Authors:** Luca Rovetini, Gianina Florentina Belciug, Luisa Massai, Francesca Nonne, Renzo Alfini, Heena Ranchod, Denasha L. Reddy, Mariagrazia Molfetta, Davide Oldrini, Makrina Totsika, Miren Iturriza, Ziyaad Dangor, Carlo Giannelli, Shabir A. Madhi, Francesca Micoli, Martina Carducci, Omar Rossi

**Affiliations:** 1 GSK Vaccines Institute for Global Health (GVGH), GSK Global Health Vaccines R&D, Via Fiorentina 1, 53100 Siena, Italy; luca.x.rovetini@gsk.com (L.R.); gianinaflorentina.x.belciug@gsk.com (G.F.B.); luisa.x.massai@gsk.com (L.M.); francesca.fn.nonne@gmail.com (F.N.); renzo.x.alfini@gsk.com (R.A.); mariagrazia.x.molfetta@gsk.com (M.M.); davide.x.oldrini@gsk.com (D.O.); makrina.x.totsika@gsk.com (M.T.); miren.x.iturriza@gsk.com (M.I.); carlo.x.giannelli@gsk.com (C.G.); francesca.x.micoli@gsk.com (F.M.); martina.x.carducci@gsk.com (M.C.); 2 WITS Vaccines and Infectious Diseases Analytics Unit, South African Medical Research Council, Faculty of Health Sciences, University of the Witwatersrand, Johannesburg 2193, South Africa; heena.ranchod@wits-vida.org (H.R.); denasha.reddy@wits-vida.org (D.L.R.); ziyaad.dangor@wits-vida.org (Z.D.); shabir.madhi@wits.ac.za (S.A.M.)

**Keywords:** *Klebsiella pneumoniae*, vaccine, immunogenicity, IgG quantification, multiplex assay, Luminex

## Abstract

*Klebsiella pneumoniae* is a leading cause of nosocomial infections, neonatal sepsis, and childhood mortality worldwide. A drastic rise in antibiotic-resistant isolates poses an urgent threat to humanity, and the World Health Organization (WHO) has classified this as a critical-priority antimicrobial-resistant (AMR) pathogen. Recent advancements in developing vaccines against *Klebsiella pneumoniae* have highlighted the lack of standardized assays to evaluate immunogenicity, complicating comparison among different vaccines under development and the establishment of a serological threshold of risk reduction (SToRR). Here, we describe the development of a ten-plex multiplex assay to measure IgG against capsular polysaccharides (K2, K25, K102, K149), O antigens (O1v1, O1v2, O2v1, O2v2 and O5), and a conserved protein (MrkA). A standard curve was established by pooling human sera from naturally exposed subjects and then calibrated in terms of Relative Luminex Units/mL. The assay was fully characterized in terms of specificity, precision, linearity, and repeatability. This immunoassay demonstrates performance suitable for future clinical trials, as well as to perform sero-epidemiological studies to gain insights into naturally occurring immunity, potentially contributing to the establishment of a serological threshold of risk reduction against *Klebsiella pneumoniae*.

## 1. Introduction

Klebsiella pneumoniae is a leading cause of nosocomial infections, neonatal sepsis, and childhood mortality globally [1,2]. A drastic rise in antibiotic-resistant isolates calls for urgent alternative strategies, and the World Health Organization (WHO) has classified Klebsiella pneumoniae as a critical-priority antimicrobial-resistant (AMR) pathogen [3], particularly in low- and middle-income countries (LMICs). Unfortunately, despite its clinical importance, a licensed K. pneumoniae vaccine is not yet available; however, several vaccines are currently under preclinical development, and a few are being tested in clinical trials [4]. Maternal vaccination would represent a valuable strategy to combat this disease. A modeling study assuming 70% coverage has suggested that a K. pneumoniae vaccine targeted at pregnant women could avert approximately 80,000 deaths and 400,000 neonatal sepsis cases, predominantly in sub-Saharan Africa and South Asia [5,6].

The bacterial surface of K. pneumoniae displays two classes of immunogenic polysaccharides, namely, capsular antigens (KAgs) and O antigens (OAgs). K antigens may represent effective vaccine targets, but their diversity (>180 different serotypes identified so far) as well as the geographical and longitudinally potential variability make the development of a capsular based vaccine challenging and require high valency [7,8,9]. There is less data available on O-antigen serogroups, although four of them (O1, O2, O3, and O5) account for >80% of clinical isolates worldwide, making an O-antigen-based vaccine attractive. However, anti-O-antigen antibodies may not offer protection in the presence of capsular polysaccharides [7,10,11,12,13,14,15]. MrkA is a key protein in the type III fimbriae complex, located on the surface of *Klebsiella pneumoniae*, playing a crucial role in attachment to host cells and biofilm formation [16]. The components of fimbriae have potential as vaccine candidates, and current vaccine development efforts are exploring various K. pneumoniae virulence factors, including outer membrane proteins, MrkA type III fimbriae, and siderophores, which are still in the early development stages [4,17].

Despite some progress in vaccine development in recent years, the lack of fully standardized assays to measure immunity to K. pneumoniae may slow down advancement. Determination of vaccine efficacy through a Ph3 clinical trial could be challenging for Klebsiella, requiring the vaccine to be administered to a very large population. Alternatively, the serological threshold of risk reduction (SToRR) can be used throughout preclinical and clinical development to predict the ability of the vaccine to generate a protective immune response. A mechanistic serological threshold of risk reduction that measures an in vitro functional response can be used; however, measuring the amount of antigen-specific binding IgG may be sufficient considering the higher complexity of functional assays [18,19]. Although several mechanisms can be triggered by antibodies, two assays have been traditionally used to look at functionality in vitro: the opsonophagocytic assay (OPK), mediated by Fc receptors in phagocytic cells and complement [20], and the serum bactericidal assay (SBA), solely mediated by complement [21]. The latter has been shown to correlate with protection mediated by anti-K antibodies in preclinical models [15]. Recently, a multiplex binding assay as well as assays to look at functional activity against O antigens have been developed [22]. However, a human reference standard and harmonized assays to assess vaccines’ immunogenicity in humans are still lacking.

Indeed, an *SToRR* for Klebsiella-mediated neonatal sepsis is not available yet, but evidence of the protective ability of anti-K. pneumoniae antibodies could be gained from case–control studies. In the specific case of neonatal sepsis, the sera of infants and/or cord blood and matched maternal sera with known infection history can be tested to measure the relative quantity of antigen-specific antibodies present and to assess their ability to kill strains with a specific KAg/OAg type. After applying a similar approach to Group B Streptococcus, it has been possible to demonstrate that the presence of a certain level of serotype-specific anti-capsular IgG was associated with a reduced risk of invasive disease among neonates [18].

With this aim, matched case–control samples coming from two different African regions (Kenya and South Africa) will be tested in binding assays and a serum bactericidal assay in a first attempt to investigate the association between antigen-specific antibody levels, and eventually their bactericidal activity, and the risk reduction of invasive Klebsiella pneumoniae disease.

In preparation for this study, we describe the setup of a multiple-bead-based assay as well as the generation and calibration of a human standard serum against several K types planned on being investigated in a case–control study (K2, K25, K102, and K149) and related O antigens (the galactose-based O-antigens O1v1, O1v2, O2v1, and O2v2 associated with K2, K102, and most of K149, and the O5 associated with K25 serotypes) plus the conserved protein MrkA. This assay was characterized using adult convalescent sera, confirming specificity, precision, linearity, and repeatability.

## 2. Materials and Methods

### 2.1. Antigen Purification

K2, K25, K102, K149, O1v1, O1v2, O2v1, O2v2, and O5 were purified from *Klebsiella* isolates expressing different KAg and OAg types, as previously reported [23]. *Kp* strains expressing K2 (5765B) and K102 (12641B) collected from the BARNARD study and the strain expressing K149 from the WITS Vaccines and Infectious Diseases Analytics Unit (WITS VIDA) collection (HG07594876B) were provided by Statens Serum Insttut (SSI). A strain expressing K25 (NCTC9145) was purchased from the PHE (Public health England) collection.

For KAg extraction, strains were plated on Worfel–Ferguson agar plates and incubated overnight (O/N) at 30 °C. After growth, bacteria were collected with a solution of 1% AcOH, and the suspension was incubated for 6 h at 100 °C in a preheated thermoblock heater. The material was then centrifuged (4000 rpm, 30 min, 4 °C), and the polysaccharide-containing supernatant was collected and filtered through 0.22 µm membranes. By fractional precipitation with cetrimonium bromide (CTAB, 102343; Merck, Darmstadt, Germany) after removal of DNA, KAg was recovered and redissolved in 1 M CaCl_2_. The KAg was then precipitated with ethanol and redissolved in water, and low-molecular-weight impurities were further removed using an Amicon 10K ultracentrifugal filter unit (UFC9010; Millipore, Burlington, MA, USA).

For OAg extraction, *Klebsiella pneumoniae* Generalized Modules for Membrane Antigens (GMMAs) were produced. O1v1, O1v2, O2v1 and O2v2 were extracted and purified from GMMA generated by Δ*tolR* mutants of the parental strain NCTC11228. For O5 GMMA, Δ*tolR* mutants of the parental strain NCTC9181 were generated. Briefly, strains were incubated O/N in LB medium at 30 °C under shaking at 180 rpm. The following day, the bacterial suspension was diluted to an optical density at 600 nm (OD_600_) of 0.05 in 50 mL of fresh LB medium and incubated during the day under the same conditions. Finally, the cultures were diluted to an OD_600_ of 0.05 in 700 mL of HTMC medium (15 g/L glycerol, 30 g/L yeast extract, 0.5 g/L MgSO_4_, 5 g/L KH_2_PO_4_, 20 g/L K_2_HPO_4_) and incubated O/N at 30 °C under shaking at 180 rpm. For each step, 50 µg/m kanamycin was added to the culture medium as a selection antibiotic. After incubation, the samples were centrifuged (45 min, 4000 rpm, 4 °C), and the supernatants were collected and filtered using 0.22 µm membranes (SLGP033R; Millipore, Burlington, MA, USA). GMMA was purified from the culture supernatant by 300K tangential flow filtration (TFF, polyethersulfone, 3081467902E--SW; Sartorius, Göttingen, Germany) with diafiltration against PBS. OAg was extracted from purified GMMA by acid treatment (acetic acid 1%, 2 h, 100 °C), and the polysaccharide was then purified by 10K TFF (Hydrosart, 3081443902E—SW; Sartorius, Göttingen, Germany) with diafiltration against 1 M NaCl and then against water. Next, sodium acetate at pH 3.7 was added to the solution to precipitate protein, and the supernatant was further purified from the residual protein using cationic exchange (CEX) filter (Sartobind S MA75, 93IEXS42DB-12—V; Sartorius, Göttingen, Germany). After pH neutralization, anion exchange chromatography with a HiTrap Q FF column (17531901; Cytiva, Chicago, IL, USA) was applied to the solution. A second 10 K TFF was finally performed on the purified OAg solution for buffer exchange.

The sequence encoding MrkA and a His-tag to simplify the purification step was inserted in pET-29b plasmid, then transformed in *E. coli* BL21 (DE3) cells (C2527H; New England Biolabs Inc., Ipswich, MA, USA). After transformation, cells were grown in HTMC until 2.0 OD/mL; protein expression induction was assessed by adding 1 mM IPTG and decreasing the growth temperature to 18 °C for 16 h. At the end of the growth period, cells were harvested and then lysed through sonication. Recombinant MrkA protein in the soluble supernatant was obtained through a two-step purification method: a Nichel affinity column followed by Superdex200 SEC (28990944; Cytiva, Chicago, IL, USA). MrkA was finally characterized by SDS-PAGE to check purity and Western blot to check binding with specific mAbs.

### 2.2. Antigen Coupling to Magnetic Beads

Biotinylation of *Klebsiella pneumoniae* purified KAg and OAg was performed through polysaccharide activation by 1-cyano-4-dimethylaminopyridinium tetrafluoro borate (CDAP) chemistry, as previously described [24,25]. The biotin hydrazide was added to the reaction mixture in excess with respect to the measured cyanilated PS sites, without isolation of the intermediate activated polysaccharide. The final biotinylated antigens were purified through two consecutive HiPrep G25 Desalting columns in an Akta system (GE17-5087-01; Cytiva) against 1 M NaCl and water, separately, to remove excess reactants. Polysaccharide quantification was performed by a phenol sulfuric assay, and the % of biotinylation was measured using 4-hydroxyazobenzene-2-carboxylic acid (HABA)/avidin reagent (H2153-1VL; Sigma-Aldrich, St. Louis, MO, USA) by its change in absorption at 500 nm, due to the displacement of HABA on avidin as mediated by biotin. Purity of polysaccharidebbiotinylated antigens and MrkA was measured by HPLC. MrkA recombinant protein and streptavidin (434301; Thermo Fisher, Waltham, MA, USA) were directly coupled to carboxylated MagPlex magnetic microspheres (Luminex Corporation, Austin, TX, USA) following the carbodiimide protein coupling method [26]. Briefly, using magnetic separation, the microspheres were washed with deionized water and resuspended in an activation buffer of 100 mM, pH 6.2, monobasic sodium phosphate (447982500; Sigma-Aldrich, St. Louis, MO, USA) and activated with an equal volume of 50 mg/mL sulfo N-hydroxysulfosuccinimide (24510; Thermo Fisher, Waltham, MA, USA) and 50 mg/mL 1-ethyl-3-(3-dimethylaminopropyl) carbodiimide hydrochloride (E2247; Thermo Fisher). Activated beads were washed twice with 50 mM, pH 5.0, 4-morpholineethanesulfonic acid (MES, M2933; Sigma-Aldrich, St. Louis, MO, USA), then incubated with the protein antigen or streptavidin for 2 h at room temperature under rotational inversion. Coupled microspheres were then washed twice with PBS (phosphate-buffered saline) with 0.05% Tween (PBST, 524653; Calbiochem, San Diego, CA, USA) and stored in PBST with 0.5% bovine serum albumin (A7030; Sigma-Aldrich) until use.

Biotinylated KAg and OAg were put in reaction with magnetic beads coupled with streptavidin. KAgs were directly mixed with commercial High Capacity Streptavidin Magplex-C microspheres (Radix BioSolutions, Georgetown, TX, USA), while, for OAg, carboxylated MagPlex magnetic microspheres were previously coupled with streptavidin, as reported above. Briefly, after two washes in PBST, microspheres coupled to streptavidin were incubated with biotinylated antigen for one hour at room temperature with rotational inversion and protected from light. Beads were then washed twice in PBST and resuspended in 1 mL of storage buffer (PBST with 0.5% BSA, A7030; Sigma-Aldrich, St. Louis, MO, USA). Each antigen was coupled to a microsphere set, identifiable through its unique spectral signature. Beads were stored in low-binding Eppendorf tubes covered from light and were well resuspended before use.

To ensure optimal coupling performance, we identified saturated conditions through the assessment of various antigen quantities: 1, 10, 60, and 100 µg/1.25 × 10^6^ beads for polysaccharide antigens, and 1, 15, and 30 µg/1.25 × 10^6^ beads for MrkA antigen. Additionally, in order to identify the minimum antigen amount necessary to maximize signal while minimizing background interference, hyperimmune serum was tested using secondary antibody at three different concentrations (from 5 to 10 µg/mL) and four different incubation times (from 15 min to 1 h).

### 2.3. Human Sera Samples

Fifteen convalescent sera from adults who had been hospitalized for invasive *Klebsiella pneumoniae* disease collected at 30–60 days after discharge from the hospital were obtained from WITS-VIDA (KPID study registered on the South African National Health Research Database reference number: GP202209026), and commercial human sera from adults (Sigma-Aldrich Code S7023 lot #SLCF9666 and H6914 lot #SLCQ0147) were screened to look at reactivity against the various antigens. High-responder sera were selected (the signal for one or more KAgs, OAgs, and/or MrkA was above 10,000 MFI) and pooled in order to achieve a high and well-balanced response among all the antigens. The pool was aliquoted, stored at −80 °C, and used as standard sera.

### 2.4. Assay Procedure and Data Analysis

A total of 50 µL of diluted standard or sample sera was mixed with 10 µL of conjugated microsphere containing 2500 beads/region/well in a 96-well Greiner plate (655904; Greiner Bio-One, Kremsmünster, Austria). Plates were incubated for 60 min at room temperature in the dark on a plate shaker at 750 rpm. After incubation, the microspheres were washed three times with 200 μL of PBS. Each well was loaded with 50 μL of 10 μg/mL (1:50 dilution) R-phycoerythrin AffiniPure goat anti-human IgG, Fcγ-fragment-specific (Jackson Immunoresearch code 109-116-098) in PBS, then incubated for 60 min in the dark under shaking at 750 rpm. After incubation, microspheres were washed three times with 200 μL of PBS, then resuspended in 100 μL of PBS. Data were acquired by Bioplex Manager 6.2 Software (Biorad, Watford, UK) using a Bioplex-200 reader, reading at least 50 beads per region at a high RP1 target. An 11-point, 3-fold serially diluted standard serum prepared in 10 mM PBS pH 7.2 was run in duplicate for all plates. Two blank wells containing PBS only were included in each plate. Test serum samples were prepared at 4 dilution points in 3-fold serial dilutions.

A 5PL parameter logistic curve was fit to the blank-subtracted median fluorescence intensity (MFI) values obtained for each standard curve point using Bioplex manager software. Assessment of the quality of the standard curve was performed using the standards recovery method by Bioplex manager Software Version 6.2 (Biorad, Watford, UK), in which the calculated (observed) concentration of each standard was compared to the nominal concentration (observed concentration/nominal concentration × 100), assigning as acceptance criteria a 30% fluctuation.

Samples were run at four different serial dilutions; the Relative Luminex Units/mL (RLU/mL) for each dilution of samples were obtained by interpolation of the blank-subtracted MFI values at each specific dilution against the 5PL parameter standard logistic curve multiplied for the initial dilution. Only values falling within the dynamic range of the fitted standard curve were considered for the analysis (automatically kept by Bioplex manager Version 6.2, Biorad, Watford, UK). Interpolation of the standard curves allowed the blank-subtracted MFI values for samples to be converted to RLU/mL. Several QC criteria were applied both to the standard and the sample. The plate was considered valid if the mean of the %relative error of the observed concentration of each calibrator of the standard curve calculated respective of the nominal concentration of the calibrator was equal or less then 20% and if the RLU/mL of two control samples fell within the expected range. The concentration in RLU/mL for each sample was calculated as the geomean of the values of the dilutions (at least two) tested for which the %relative error of each dilution versus the median of the RLU/mL of the four dilutions was within the 30%. The sample was considered valid if the %CV of valid geomeans were equal or less then 30%.

### 2.5. Assay Performance Characterization

Upper and lower limits of standard curve accuracy (ULSCA and LLSCA) were determined by assaying sixteen independent standards run in multiple days. Average RLU/mL values of standard curves were used to fit 5PL regression curve to MFI data. Residual error (RE%) was calculated against the nominal concentration of the standard curve using the formula

$$Residual\;Error\;(ER\%)=\frac{Observed\;RLU/mL-Nominal\;RLU/mL}{Nominal\;RLU/mL}\cdot 100$$

The lower and upper limits of standard curve accuracy (LLSCA, ULSCA) were set, where the predicted RE% with 90% confidence was within ±25%. The lower limit of quantification (LOQ) was the LLSCA multiplied by 100.

Monoplex versus multiplex performance was assessed by running the same sera in parallel both in monoplex (where a single-antigen-coupled microsphere region was incubated with the test samples) and multiplex (where all ten antigen-coupled microsphere regions were incubated simultaneously with test samples) in the standard assay.

Repeatability (R) and intermediate precision (IP) were assessed by testing six replicates of three human sera (standard serum, and two commercial sera #S7023 #H6914) across four different sessions conducted by the same operator. Each sample was prepared and evaluated at four different dilutions (3-fold apart) using the standard assay. Analysis of variance (ANOVA) was conducted on log10-transformed RLU/mL for each of 24 separate tests for each serum. Coefficients of variance for repeatability and intermediate precision were then calculated from the log-transformed variance components and converted back to original RLU/mL units using the equation CV = √(exp(sLn^2^) − 1), where sLn is calculated as the standard deviation of log10-transformed variance components multiplied by Ln (10).

Assay linearity was evaluated by testing human standard serum, either neat or diluted in PBS, in six 2-fold serial dilutions, 1:2, 1:4, 1:8, 1:16, 1:32, and 1:64, prior to performing the assay. The linear regression between the observed log10-transformed RLU/mL and the log10 nominal RLU/mL, corrected by the dilution factor, was determined. The R^2^ value was calculated, along with the confidence intervals (CIs) for both the slope and the y-axis intercept.

Assay specificity: Human standard serum was diluted 1:1 (v:v) in PBS alone or PBS supplemented with 11 different quantities (from 30 to 0.0005 µg/mL) of different antigens. Plates were incubated for 1 h at room temperature, shaking at 600 rpm before adding the 10-plex beads and continuing with the standard assay. The percentage of inhibition at each concentration of the competitor was determined by comparing it with an uninhibited sample under the same conditions for each antigen-coupled microsphere region using the following formula:

$$Inhibition\;\%=\frac{RLU/mL\;Control-RLU/mL\;Inhibited\;sample}{RLU/mL\;control}\cdot 100$$

The amount resulting in ≥70% homologous inhibition was then used to assess the percentage of the heterologous inhibition.

The limits of the blank were assessed by testing 18 replicates of an IgG-depleted sera (Molecular Innovations Code HPLA-SER-GF #HPLA-SER-GF-121) in two independent runs as the test sample.

## 3. Results

### 3.1. Assay Setup, Calibration of Standard Sera and Multiplexing

In order to quantify the level of anti-KAg, anti-OAg and anti-MrkA antibodies in human sera, a 10-plex serological Luminex assay was developed. The different antigens were purified,; the polysaccharides were biotinylated and fully characterized (Table 1).

Ten different MagPlex microsphere sets were coupled to purified antigens. Biotinylated polysaccharides K25 had a residual nucleic acid content of <5% while for the other biotinylated polysaccharides, the residual nucleic acid content was <1%. HPLC traces of biotinylated material did not evidence the presence of residual protein. For MrkA, a purity of >98% was confirmed.

Different amounts of KAg (ranging from 1 to 100 µg/1.25 × 10^6^ beads), OAg (ranging from 1 to 100 µg/1.25 × 10^6^ beads), and MrkA (ranging from 1 to 30 µg/1.25 × 10^6^ beads) were tested to identify the optimal bead coupling conditions. The anti-human IgG detection antibody was titrated as well in terms of dilution and incubation time. Conjugation concentrations of 1 µg/1.25 × 10^6^ beads for KAg and OAg and 15 µg/1.25 × 10^6^ beads for MrkA, with a phycoerythrin-labeled secondary antibody at concentration of 10 μg/mL, were chosen to have the maximal dynamic range for all antigens: maximal mean fluorescence intensities >20,000 with concentrated positive sera and low background (blank) MFIs (result of <150 MFI) with assay buffer alone.

**Figure 1:**
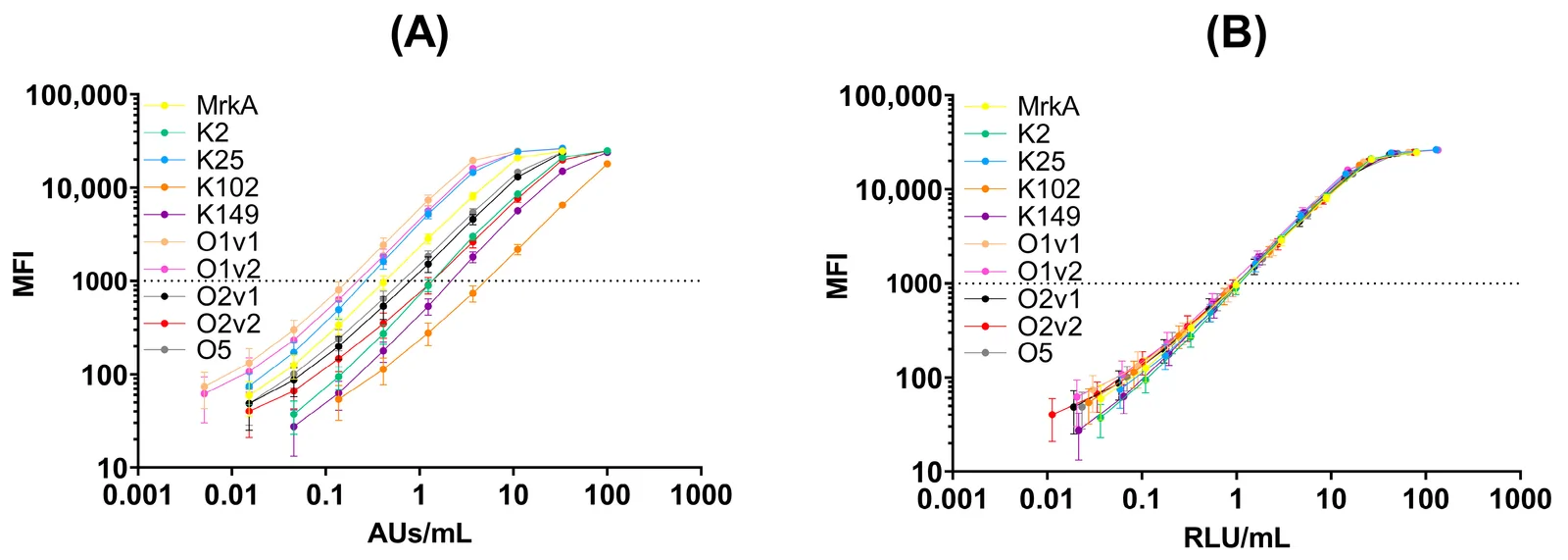
Standard curve of MFI versus arbitrary units (AUs) obtained against each specific antigen pre-calibration (**A**) and in RLU/mL post-calibration (**B**). Geomean of individual points and standard deviation are shown for each datapoint together with the 5PL logistic curve fit; 1 RLU/mL was defined as the reciprocal sera dilution giving a signal of 1000 MFI (dotted line) in a standard assay.

The convalescent sera were screened for their positivity to the different antigens to generate a hyperimmune standard serum. The newly generated serum confirmed positivity against all antigens coupled to beads (Figure 1A).

In the multiplex immunoassay, the same serum was run at the same dilution; therefore, unless generated by pooling the equivalent amount of antigen-specific antibodies (condition possible at preclinical level but not often in humans), the 5 PL curve fitted to MFI values obtained at the different dilutions resulted in a different reactivity against the different antigens (Figure 1A). However, by defining 1 RLU/mL as the reciprocal of the sera dilution that gave 1000 MFI in a standard assay, it was possible to calibrate the standard curve against each antigen taking into account the reactivity in the assay with respect to the initial sera dilution in the assay (Table 2).

Thus, through this normalization, we obtained a standard curve showing a similar dynamic range against each antigen that was suitable for use in a fully quantitative assay, also allowing the relative comparability of the response between different antigens (Figure 1B).

The stability of antigens attached to the beads was verified for a minimum of 4 months by assessing the reactivity of the standard sera against various antigens at multiple timepoints. In all instances, equivalent performances were observed, as indicated by overlapping curves at different timepoints (Supplementary Figure S3). Additionally, two separate batches of beads were coupled to antigens, the MFIs obtained were confirmed to be consistent across all antigens between the two bead batches (Supplementary Figure S2), and same was true for the RLU/mL obtained with the quality control samples. Those data confirmed the ability of the assay to perform in equivalent manner for months, a critical characteristic when larger trials have to be run, as well as the negligible impact of testing with different beads lots.

**Figure 2:**
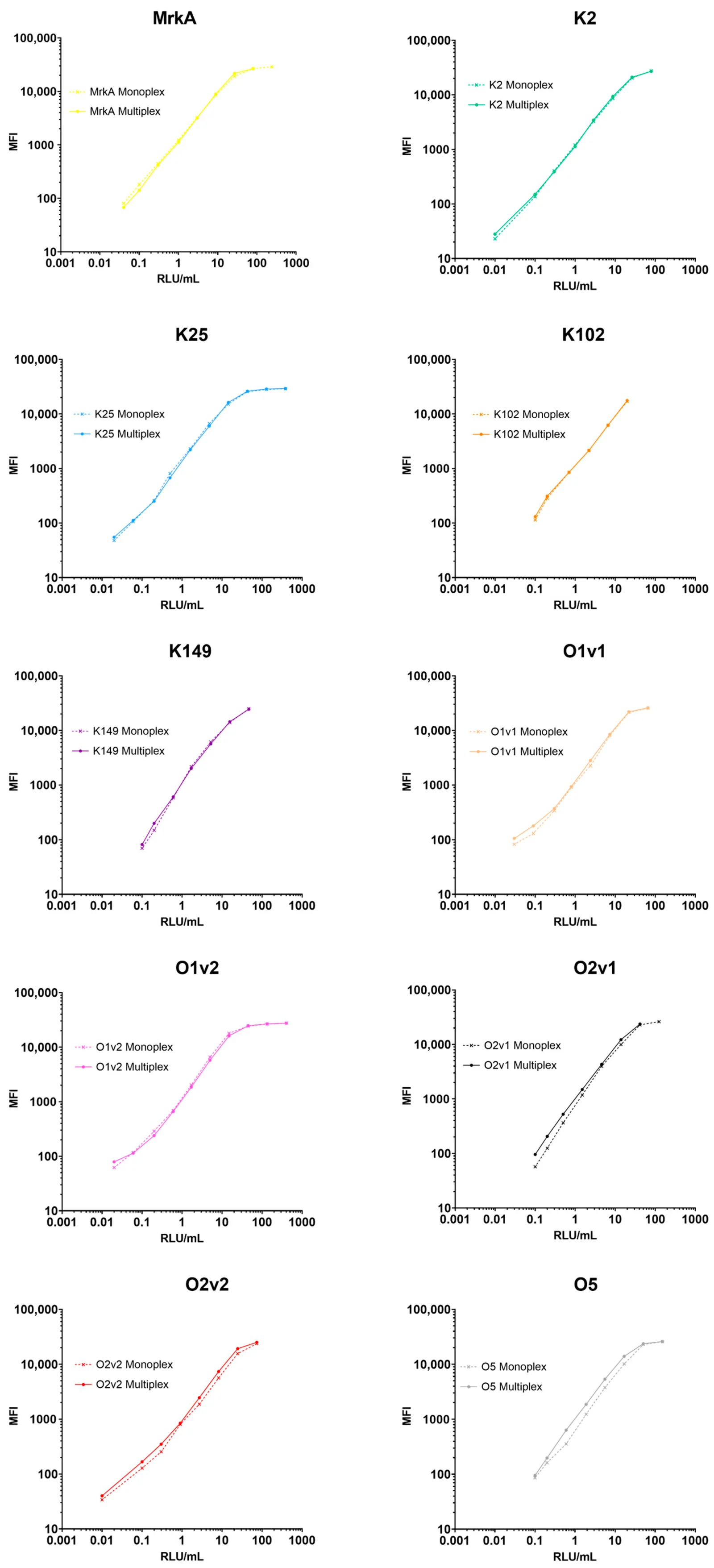
Monoplex and multiplex standard curves: multivalent human standard serum was incubated with single (dotted line) or multiplex (continue line) bead preparations, and the MFIs were compared.

To evaluate the effects of serotype cross-reactivity and the influence of multiplexing, the MFI of multivalent standard serum in the 10-plex assay was compared to that obtained from an assay using single-plex beads. The MFIs of the multivalent standard serum in both the 10-plex and single-plex assays were similar for all antigens, as evidenced by the overlapping curves in both settings (Figure 2).

The lower and upper limits of standard curve accuracy were established for all antigens (Table 2). Each antigen’s dynamic range exceeded 2 logs, with O1v1 having the smallest range, still spanning 140-fold. This offers the possibility of allowing dilutions of the sera to be quantified easily, falling within the dynamic range of the standard curve. Also note that, since multiple dilutions of the same serum were tested, the results were obtained from multiple datapoints falling within the quantifiable range of the standard curve, thus obtaining optimal accuracy with the minimal amount of retests.

The limit of quantification for the assay was established by multiplying the limit of the standard curve accuracy by 100, which was the lowest sera concentration tested in this layout. Since the assay has no virtual upper limit of quantification, samples at the tested dilutions that do not fall with the accuracy range of the standard curve can be retested at higher dilutions.

Thus, we established a standard curve with good accuracy and dynamic range, which can be executed at 11 different dilutions alongside a duplicate buffer-only well (blank) on each plate. Up to 16 different sera plus two control samples with known reactivity can be run at four distinct dilutions and MFI values interpolated against the standard curve. Results are presented by the geomean of all RLU/mL obtained for the dilution falling within the dynamic range of the standard curve.

### 3.2. Assay Performance: Precision, Linearity, and Specificity

**Figure 3:**
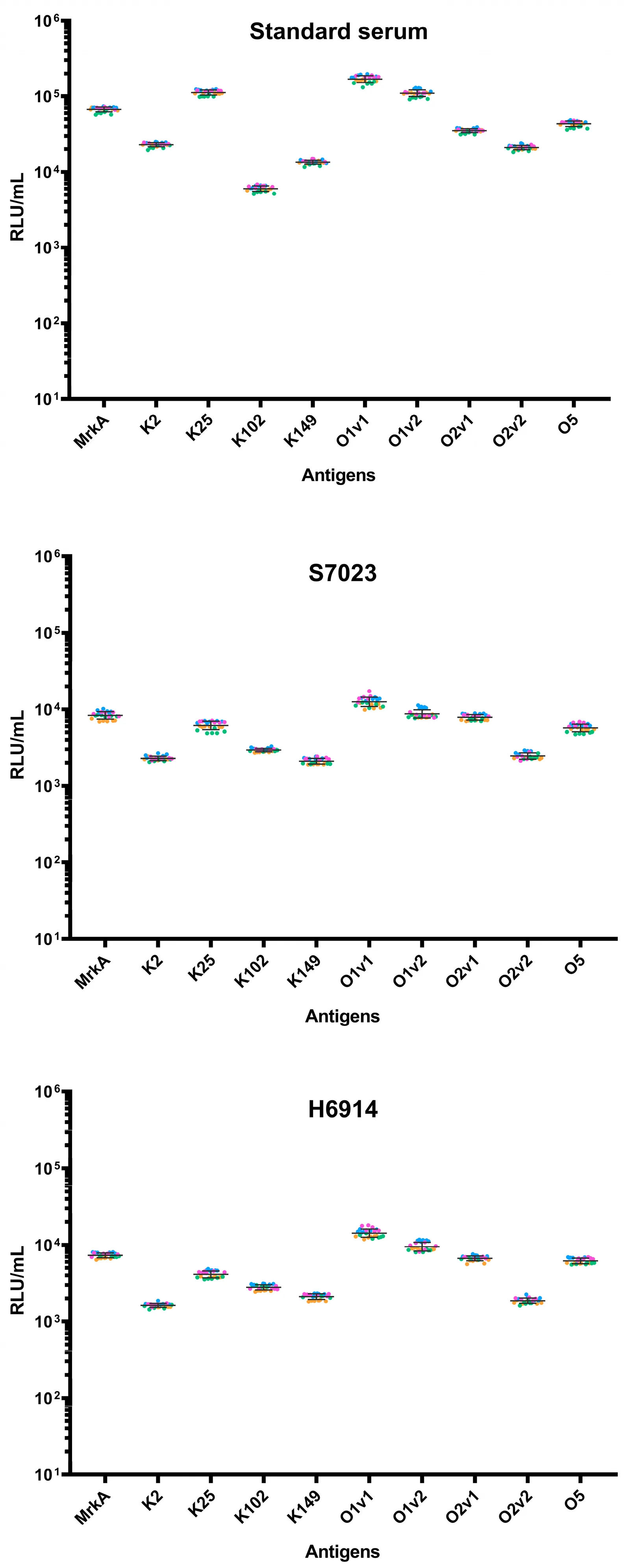
Assay precision. A total of 24 repeated measurements of RLU/mL from single independently handled samples by one operator in four different experiments. Single repeats are represented by circle symbols (repeats on different days are shown in orange for run 1, green for run 2, blue for run 3, and pink for run 4). Geometric means and geometric standard deviations from all repeats of each sample are represented by the grey line.

After having established the assay layout and the standard curve, assay precision was assessed testing six replicates of three human sera in four different experiments (Figure 3).

The multiplex assay has an overall repeatability of <6.5% and intermediate precision of <13.6% (Table 3), and the analysis session did not influence assay variability (*p*-values > 0.05 in all cases).

When testing the negative matrix in the standard assay, the MFI detected was <LLSCA at a 1:100 dilution for all the antigens, thus suggesting the strong ability of the assay to discriminate between negative and positive samples.

**Figure 4:**
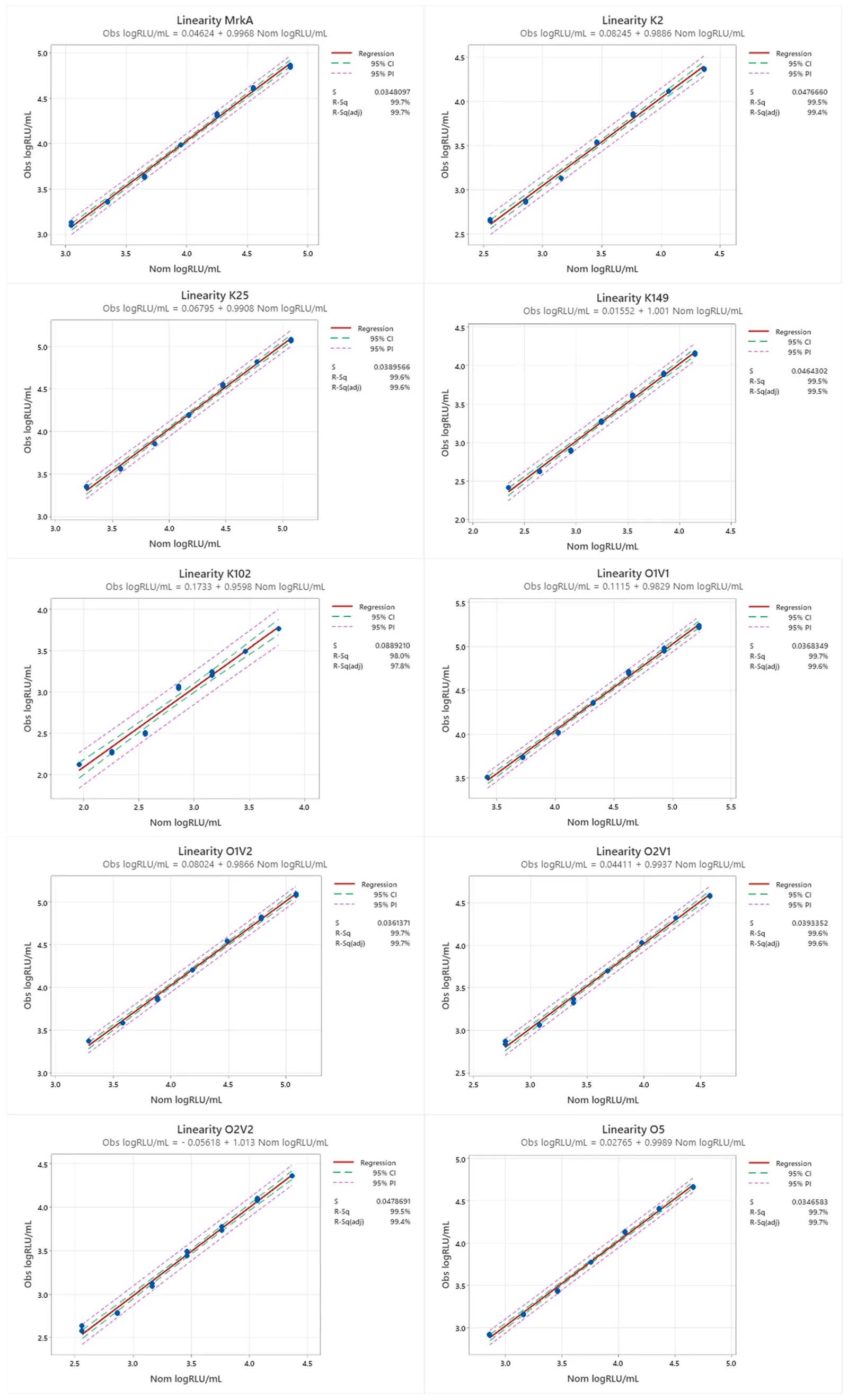
Assay linearity regression analysis. Blue dots represent log(RLU/mL) obtained for specific diluted sera. Red line represents the linear regression trendline, and 95% CI and 95% PI are also reported.

When linearity was assessed by running a pre-diluted sample in a standard assay, the linear regression models comparing the obtained values to the expected ones showed intercepts not significantly different from 0 and slopes not significantly different from one (Table 3 and Figure 4), confirming the linearity of the assay for all the different antigens.

Finally, we determined the specificity of the assay by depleting human sera with different concentrations of each homologous competitor antigens prior to testing in the standard assay. The lowest concentration of the homologous competitor able to inhibit the signal by at least 70% was determined for each antigen and used to establish heterologous specificity (Table 4).

Good specificity was confirmed for all antigens, as percentage inhibition was >70% after incubation with homologous competitors and generally lower than 30% for each heterologous antigen. As expected, a certain level of cross-reactivity was observed in the presence of certain heterologous competitors like different versions of galactose-based OAg.

## 4. Discussion

Here, we describe the setup and detailed characterization of a ten-plex multiplex assay to measure IgG in human sera against ten *Klebsiella pneumoniae* antigens KAgs (K2, K25, K102, K149) and OAgs (O1v1, O1v2, O2v1, O2v2 and O5) as well as conserved MrkA protein, using a bead-based assay based on Luminex technology. Compared to traditional ELISA techniques, multiplex assays require smaller sample volumes, reduces the analysis time, and minimize the use of reagents. This makes them ideal for cost-effective, high-throughput applications in large-scale vaccine studies, particularly in case of multivalent vaccines, such as those targeting *Klebsiella pneumoniae*. Luminex technology has been already used to develop serology assays targeting bacterial antigens, including the validated pneumococcal capsular polysaccharide assay [27,28].

We demonstrated that the multiplex assay exhibits specificity, with notable cross-reactivity primarily occurring between OAg antigens, as expected, due to the antigens’ structural similarity [22,29]. Occasional cross-reactivity was also observed between KAgs and OAgs, although the inhibition was weaker compared to homologous antigens. This cross-reactivity is unlikely to be attributed to antibodies targeting impurities, given the high purity of the antigens used in the assay. Confirmation of this cross-binding further supports the potential for cross-protection following natural infection and/or vaccination. However, the detected binding must be critically compared with that observed in the presence of homologous binding, as well as the avidity of the antibodies and their ability to induce appropriate functional effector functions, all of which need to be carefully assessed in clinical trials. Furthermore, the assay is precise, repeatable, and performs linearly across a large concentration range. The dynamic range of the standard curve spans within at least 2 logs of dilutions (reaching 3 log for some of the antigens); thus, the assay can accurately quantify IgG titers across a large range of concentrations at a given test sample dilution. The assay showed low limits of quantification for each antigen and has no virtual upper limit of quantification; thus, the samples at the tested dilutions not falling within the accuracy range of the standard curve can be retested at higher dilutions. Moreover, only negligible variations when using antigen-coupled beads for over four months and between different production lots were observed, confirming the assay’s robustness and therefore the reliability in generating reproducible data over prolonged periods, essential for studies requiring extensive testing. Our newly developed ten-plex assay represents a significant enhancement in the evaluation of *Klebsiella pneumoniae* antigen-specific IgG responses compared to existing protocols [22,29]. Indeed, a four plex assay was previously described to quantify anti-OAg antibodies using mainly rabbit sera and the commercially available human IVIG preparation; in the current study, we confirmed the similar performance of the assay in quantifying anti-OAg IgG with naturally exposed and commercial human samples and expanded the range of antigens to include capsular polysaccharides and OAg, along with the conserved protein MrkA. A distinguishing feature of our assay and an important advancement in terms of the comparability of results in the absence of internationally calibrated standard is represented by the calibration of the standard curve. This calibration ensures that the results are consistent and comparable across various settings, addressing a major limitation of previous assays. We addressed the common challenge of varied reactivity across different antigens in multiplex assays by defining 1 RLU/mL as the reciprocal of the sera dilution yielding 1000 MFI in a standard assay, allowing for individual standard curve calibration for each antigen.

The standard curve characterization and calibration established here can be used to standardize the IgG measurement of the antigens between plates on different days and potentially to compare assays performed in different laboratories, compare different studies, and the ability of multiple standards to be bridged. Therefore, the immunoassay described here is suitable for measuring serological responses in both vaccine trials and studies of infection-induced immunity.

## Figures and Tables

**Table 1:** Antigen characterization and coupling conditions. (Mn: number of average molecular weight; Mw: average molecular weight; Mp: mode molecular weight; polydispersity: Mw/Mn).

Antigen	Mn–Da	Mw–Da	Mp–Da	Polydispersity	$\mathrm{Activation}\%\;\frac{nmol\;biotin}{nmol\;sugar\;monomers}$	**µg Ag/1.25 × 10^6^ Beads**
MrkA	N/A	N/A	57,524	N/A	N/A	15
K2	215,734	508,155	428,219	2.4	0.65%	1
K25	119,280	342,106	287,795	2.9	0.041%	1
K102	162,993	355,310	309,677	2.2	0.04%	1
K149	40,037	121,546	74,583	3.0	2.5%	1
O1V1	17,220	26,106	20,076	1.5	4.9%	1
O1V2	17,076	26,971	22,600	1.6	4.6%	1
O2V1	12,711	24,357	11,569	1.9	5.8%	1
O2V2	12,156	17,681	12,638	1.5	4.5%	1
O5	13,028	18,686	12,758	1.4	2.5%	1

**Table 2:** Standard RLU/mL, LLSCA, ULSCA, and LLOQ assigned to each Ag of human standard sera.

	MrkA	K2	K25	K102	K149	O1V1	O1V2	O2V1	O2V2	O5
Standard RLU/mL	72,010	23,857	115,697	5974	14,020	177,722	120,959	37,426	22,107	45,613
LLSCA	0.04	0.09	0.06	0.11	0.12	0.27	0.17	0.10	0.18	0.04
ULSCA	98.09	79.52	113.97	19.91	46.73	38.88	41.39	53.55	48.70	63.96
LLOQ	4	9	6	11	12	27	17	10	18	4

**Table 3:** Assay precision and linearity analysis for each antigen.

Ag	Precision		Linearity					
	Repeatability (%)	Intermediate Precision (%)	Term	Coef	SE Coef	95% CI	r^2^ (adj)	Regression Equation
MrkA	5.6	9.5	Intercept	0.0462	0.0617	(−0.0883; 0.1808)	99.7%	Obs logRLU/mL = 0.0462 + 0.9968 Nom logRLU/mL
			Slope	0.9968	0.0155	(0.9632; 1.0305)		
K2	4.1	6.7	Intercept	0.0824	0.0743	(−0.0795; 0.2444)	99.4%	Obs logRLU/mL = 0.0824 + 0.9886 Nom logRLU/mL
			Slope	0.9886	0.0212	(0.9425; 1.0347)		
K25	3.6	11.8	Intercept	0.068	0.0729	(−0.0908; 0.2267)	99.6%	Obs logRLU/mL = 0.0680 + 0.9908 Nom logRLU/mL
			Slope	0.9908	0.0173	(0.9531; 1.0285)		
K102	5.1	7.8	Intercept	0.173	0.115	(−0.078; 0.425)	97.8%	Obs logRLU/mL = 0.173 + 0.9598 Nom logRLU/mL
			Slope	0.9598	0.0395	(0.8738; 1.0458)		
K149	4.9	8.4	Intercept	0.0155	0.068	(−0.1327; 0.1638)	99.5%	Obs logRLU/mL = 0.0155 + 1.0010 Nom logRLU/mL
			Slope	1.001	0.0206	(0.9561; 1.0459)		
O1V1	6.5	13.6	Intercept	0.1115	0.0714	(−0.0441; 0.2670)	99.6%	Obs logRLU/mL = 0.1115 + 0.9829 Nom logRLU/mL
			Slope	0.9829	0.0164	(0.9473; 1.0185)		
O1V2	5.4	13.2	Intercept	0.0802	0.0679	(−0.0676; 0.2281)	99.7%	Obs logRLU/mLv = 0.0802 + 0.9866 Nom logRLU/mL
			Slope	0.9866	0.016	(0.9516; 1.0215)		
O2V1	4.5	7.9	Intercept	0.0441	0.0651	(−0.0977; 0.1859)	99.6%	Obs logRLU/mL = 0.0441 + 0.9937 Nom logRLU/mL
			Slope	0.9937	0.0175	(0.9557; 1.0317)		
O2V2	5.4	8.7	Intercept	−0.0562	0.0747	(−0.2190; 0.1066)	99.4%	Obs logRLU/mL = −0.0562 + 1.0133 Nom logRLU/mL
			Slope	1.0133	0.0212	(0.9670; 1.0596)		
O5	4.0	10.5	Intercept	0.0276	0.0586	(−0.1000; 0.1553)	99.7%	Obs logRLU/mL = 0.0276 + 0.9989 Nom logRLU/mL
			Slope	0.9989	0.0154	(0.9654; 1.0324)		

**Table 4:** Homologous and heterologous specificity reported as inhibition %. In column “Ag (µg/mL)”, the concentration of antigen used to determine the %inhibition against each specific Ag is shown. * Used rabbit polyclonal standard sera.

Ag	Ag (µg/mL)	Inhibition %										
		MrkA	K2	K25	K102	K149	O1v1	O1v2	O2v1	O2v2	O5	
MrkA	0.12	71	3	0	0	0	0	3	0	0	9	0% Inhibition 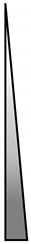 70% Inhibition
K2	0.37	2	70	31	3	1	0	17	7	0	3	
K25	0.002	0	0	71	0	0	0	0	1	0	0	
K102 *	0.005	12	1	4	72	0	10	0	1	3	9	
K149	10	2	14	0	0	70	0	31	13	0	3	
O1V1	0.12	0	32	3	0	0	77	74	21	0	25	
O1V2	0.014	0	0	2	0	0	67	72	4	0	3	
O2V1	1.11	0	9	1	0	9	38	0	75	17	3	
O2V2	30	0	0	9	26	56	0	52	1	76	0	
O5	0.12	0	0	7	0	2	1	3	0	0	78	

## Data Availability

The original contributions presented in this study are included in the article/Supplementary Material. Further inquiries can be directed to the corresponding author.

## Supplementary Materials

The following supporting information can be downloaded at: https://www.mdpi.com/article/10.3390/mps8030052/s1, Figure S1: Plate Layout; Figure S2: Two separate batches of beads were coupled to antigens, and the mean fluorescence intensities (MFIs) obtained were confirmed consistent across all antigens; Figure S3: Beads stability: mean fluorescence intensities (MFIs) obtained at different time-points were confirmed consistent across all antigens.
