# Supplementary material for: Development and Characterization of a Ten-Plex Assay to Measure Klebsiella pneumoniae Antigen-Specific IgG in Human Sera
Source: Methods Protoc. 2025 May 19;8(3):52. doi: 10.3390/mps8030052 (PMC12101422; doi:10.3390/mps8030052)
Supplement: Supplementary file 1 [file mps-08-00052-s001.zip › mps-3623205-supplementary.pdf]

Supplementary Materials:

1.1 Plate Layout

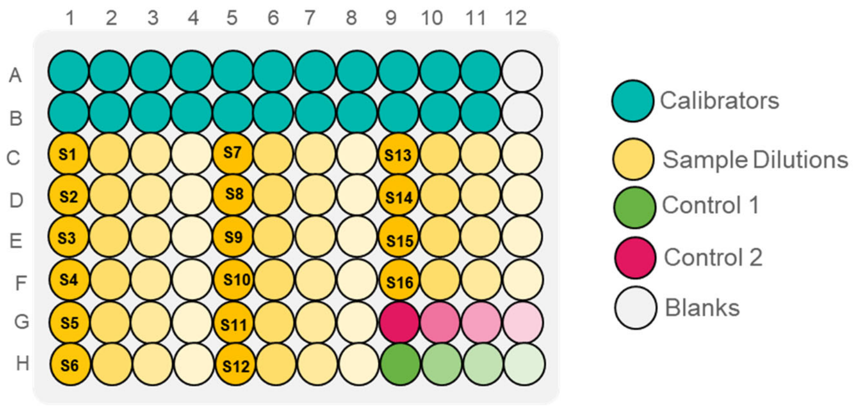

Figure S1 - Plate Layout

1.2 Beads batches comparison

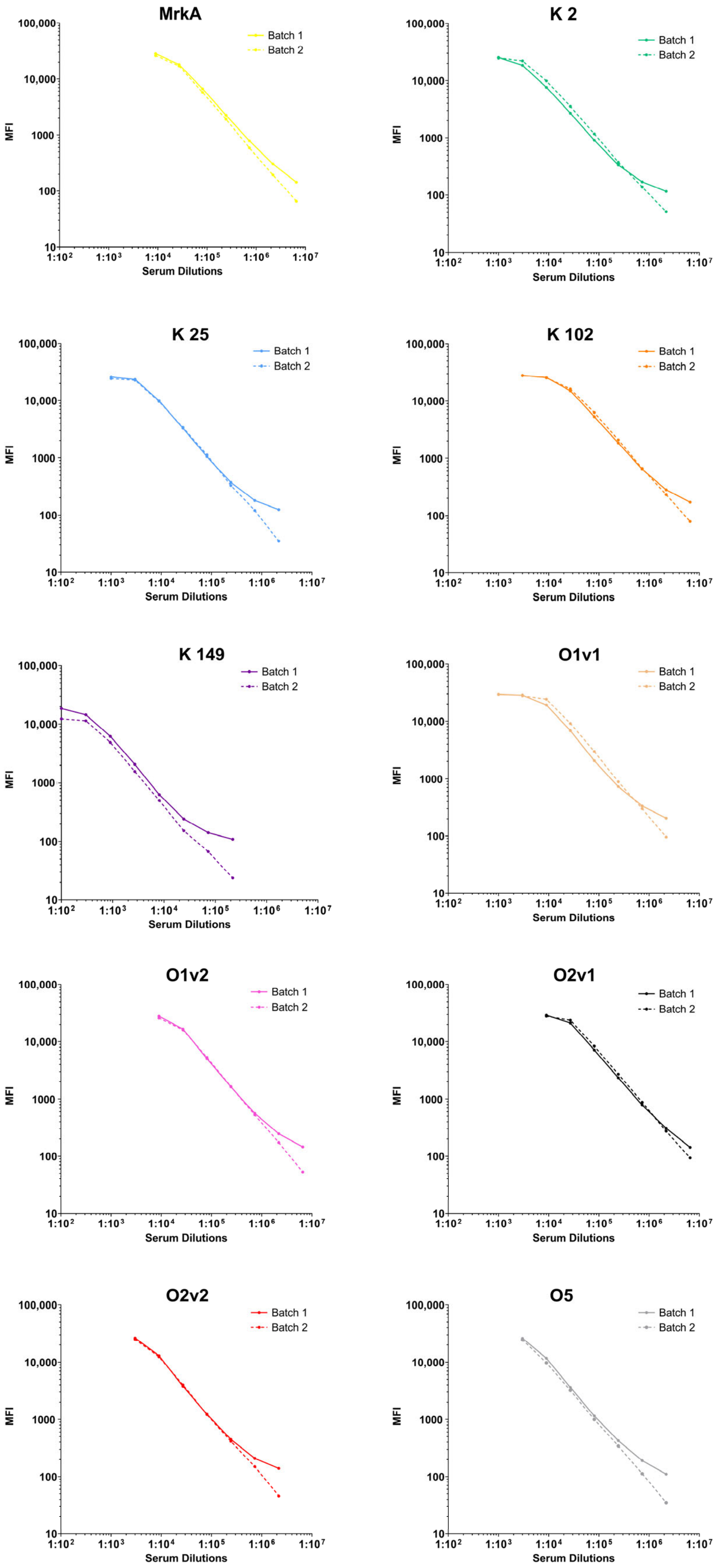

Figure S2 - Two separate batches of beads were coupled to antigens, and the mean fluorescence intensities (MFIs) obtained were confirmed consistent across all antigens.

1.3 Beads Stability

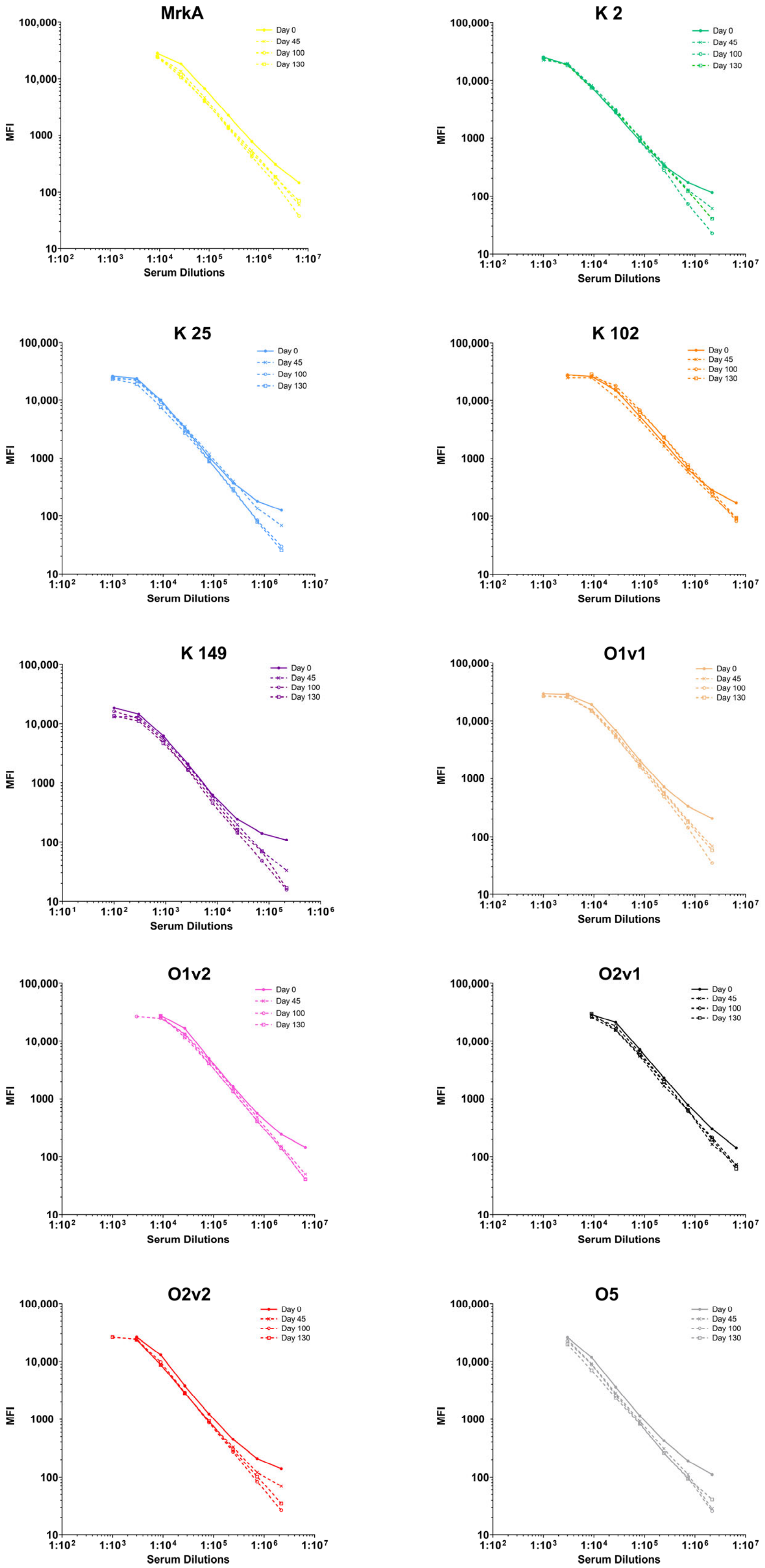

Figure S3: Beads stability: mean fluorescence intensities (MFIs) obtained at different time-points were confirmed consistent across all antigens.
